# Calcification of the elastic component: the impact on the cardiovascular system

**DOI:** 10.3389/fcvm.2025.1636812

**Published:** 2025-08-25

**Authors:** Francesco Demetrio Lofaro, Alessia Mazzilli, Susanna Bonacorsi, Daniela Quaglino, Federica Boraldi

**Affiliations:** Department of Life Sciences, University of Modena and Reggio Emilia, Modena, Italy

**Keywords:** elastic fibre, mineralization, elastocalcinosis, cardiovascular system, extracellular matrix, calcification models

## Abstract

In the cardiovascular system, elastic fibres exert a fundamental role providing the long-range elasticity required for physiological functions. Elastic fibres are complex in composition and structure containing, in addition to elastin, a wide range of matrix components, such as microfibrillar proteins, calcium-binding proteins and glycosaminoglycans. Changes in composition and/or structure can affect the biomechanics of the tissue as well as the intrinsic affinity of elastin for Ca^2+^ ions. Mineralization of elastic fibres can occur in genetic as well as in age-related chronic diseases. In cardiovascular diseases, for instance, calcification represents an integral part of the pathogenetic process, although the regulatory mechanisms are not completely understood. Therefore, a focus is given on elastin synthesis and assembly, on elastic fibre components and on elastin degradation. Moreover, the role and the impact of altered composition and supramolecular organization of elastic fibres are described in the context of the calcified cardiovascular system. Finally, some *in vitro* and *in vivo* models of elastic fibres calcification are presented and discussed.

## 1 Introduction

Elastin is the typical component of the elastic fibres and is the major responsible for soft connective tissue elasticity. The number and size of elastic fibres/lamellae are strictly related to the capacity of tissues to get back to their original shape after stretching. Indeed, elastic fibres are made of a wide range of proteins, glycoproteins and glycosaminoglycans (GAGs) whose amount and composition vary depending on the functional requirements of the tissue (1).

In vertebrates, elastin is mainly produced by fibroblasts and vascular smooth muscle cells (VSMC) during the second half of gestation until the end of childhood; thereafter the elastin gene is almost completely silent with a turnover evaluated to be approximately about 70 years (2). Therefore, elastin is one of the most resistant proteins, being present even in very old subjects, although with lower efficiency. Over the lifetime, elastin accumulates damages as the result of the aging processes and of environmental *noxae*, which cause progressive and irreversible loss of function especially in the cardiovascular system (3).

Cardiovascular calcification is an integral part of aging and of many cardiovascular diseases such as atherosclerotic microcalcifications, vascular and heart valve mineralization (4). Calcification in blood vessels occurs in the intima (in the context of atherosclerosis and in association with lipids, macrophages and VSMC), and in the media (involving elastin and VSMC). In both cases, areas of mineralization are associated with matrix vesicles and with changes in the expression of mineralization-regulatory proteins (5). Density of arterial calcification exhibit an age-dependent increase contributing to progressive vascular stiffness.

Calcific aortic valve disease comprises either aortic valve (AoV) sclerosis, with mild AoV thickening and/or calcification without obstruction of blood flow, and AoV stenosis with more severe calcification and impaired leaflet function. The intrinsic calcification mainly found at the leaflet hinge is characterized by abnormal collagen and proteoglycan (PG) deposition, whereas from the middle to the tip regions, in more severely affected leaflets and surgical specimens, nodular calcification and increased elastin fragmentation are more frequently observed (6). The elastin calcification propensity seems to directly depend on the size of elastin aggregates and the Ca^2+^ concentration allowing stronger interactions compared to collagen (7). These data may explain the prevalent occurrence of elastocalcinosis in the cardiovascular system and also in the bioprosthetic valves which are known to be at high risk of calcification and replacement after few years (8).

Calcification of the cardiovascular system depends also on the active role of many cells, such as VSMC and endothelial cells that can transdifferentiate in pro-osteogenic cells (9, 10), as well as VSMC and platelets that release extracellular vesicles enriched in pro-mineralizing factors capable to trigger calcification of the elastic component (11–13).

Since mineral deposits are not simply due to passive precipitation of calcium phosphate but are rather the result of a more complex biomineralization process, in this context, the extracellular matrix (ECM), and in particular elastic fibres are important players in the formation of ectopic calcified structures, because of their affinity for calcium ions, even in the absence of cells (14).

## 2 Elastin and elastic fibres

Elastin is the main component of elastic fibres, confers elasticity and contributes to tissue biomechanical properties of tissues such as vessels, skin, lungs as well as cartilage and intervertebral discs (15–17). Indeed, elastic fibres are composed of many proteins and glycoproteins and are structurally organized in fibres of different sizes, in larger networks or in lamellae depending on the functional requirements of the tissue.

**Figure 1:**
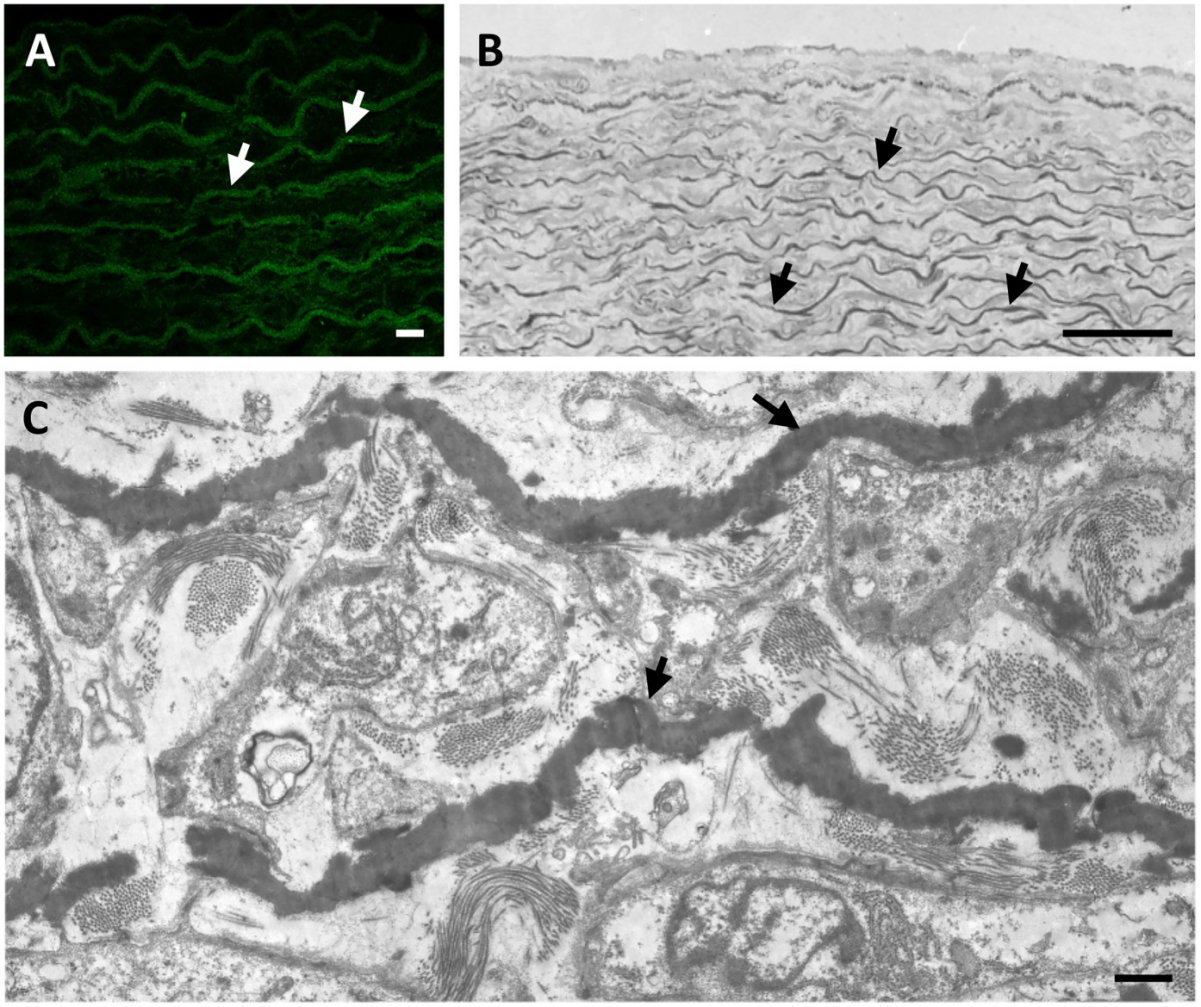
The elastic component (arrows) in arteries is typically organized in form of lamellae parallel to the lumen and alternated with smooth muscle cells in animal models such as rat **(A)** and in humans **(B,C)**. Elastic fibres in the rat aorta are shown by fluorescence microscopy after staining with haematoxylin and eosin H **(A)**. Human foetal aorta is visualized by light microscopy after toluidine blue staining of semithin sections **(B)** and by transmission electron microscopy of ultrathin sections **(C)**. Samples have been routinely processed for morphological analyses (18, 19) and figures are unpublished original images from the Authors' archive. Scale bar: 10 μm **(A)**; 100 μm **(B)**; 1 μm** (C)**.

Elastic fibres can be visualized (Figure 1) by light microscopy also using specific stains such as Verhoeff-Van Gieson or Weighert's (20), by electron microscopy (21, 22), by fluorescence and confocal microscopy after haematoxylin-eosin/phloxine staining (23) and even *in vivo* by multiphoton microscopy due to its intrinsic fluorescence properties (24).

Elastin represents approximately 90% of elastic fibres but, to date, more than 30 molecules have been shown to associate with fibres (Table 1) contributing to their formation and maintenance *in vivo* (66).

**Table 1:** Elastic fibre components and associated molecules.

Elastic fibre components				
Description	Accession^a^	Protein ID	Gene	References
Basement membrane-specific heparan sulphate proteoglycan core protein (Perlecan)	P98160	PGBM	HSPG2	(25)
Biglycan	P21810	PGS1	BGN	(26, 27)
Collagen alpha-1(VI) chain	P12109	CO6A1	COL6A1	(28)
Collagen alpha-2(VI) chain	P12110	CO6A2	COL6A2	
Collagen alpha-3(VI) chain	P12111	CO6A3	COL6A3	
Collagen alpha-6(VI) chain	A6NMZ7	CO6A6	COL6A6	
Collagen alpha-1(VIII) chain	P27658	CO8A1	COL8A1	(28)
Collagen alpha-2(VIII) chain	P27658	CO8A2	COL8A2	
Collagen alpha-1(XVI) chain	Q07092	COGA1	COL16A1	(28)
Chondroitin sulphate (CS)	–	–	–	(26, 27, 29)
Decorin	P07585	PGS2	DCN	(26, 29)
EGF-containing fibulin-like extracellular matrix protein 1 (Fibulin-3)	Q12805	FBLN3	EFEMP1	(30, 31)
EGF-containing fibulin-like extracellular matrix protein 2 (Fibulin-4)	O95967	FBLN4	EFEMP2	(30, 32–34)
Elastin	P15502	ELN	ELN	(28)
Emilin-1	Q9Y6C2	EMIL1	EMILIN1	(28)
Emilin-2	Q9BXX0	EMIL2	EMILIN2	(28)
Fibrillin-1	P35555	FBN1	FBN1	(35, 36)
Fibrillin-2	P35556	FBN2	FBN2	(35)
Fibrillin-3	Q75N90	FBN3	FBN3	(37)
Fibulin-1	P23142	FBLN1	FBLN1	(30)
Fibulin-2	P98095	FBLN2	FBLN2	(30)
Fibulin-5	Q9UBX5	FBLN5	FBLN5	(32, 38–40)
Heparan sulphate (HS)	–	–	–	(25)
Latent-transforming growth factor beta-binding protein 1	Q14766	LTBP1	LTBP-1	(41, 42)
Latent-transforming growth factor beta-binding protein 2	Q14767	LTBP2	LTBP-2	(43, 44)
Latent-transforming growth factor beta-binding protein 3	Q9NS15	LTBP3	LTBP-3	(42)
Latent-transforming growth factor beta-binding protein 4	Q8N2S1	LTBP4	LTBP-4	(42)
Lysyl oxidase homolog 1	Q08397	LOXL1	LOXL1	(45)
Microfibril-associated glycoprotein 3	P55082	MFAP3	MFAP3	(28)
Microfibril-associated glycoprotein 4	P55083	MFAP4	MFAP4	(28)
Microfibrillar-associated protein 1	P55081	MFAP1	MFAP1	(28)
Microfibrillar-associated protein 2	P55001	MFAP2	MAGP-1	(46, 47)
Microfibrillar-associated protein 5	Q13361	MFAP5	MAGP-2	(48, 49)
Osteopontin	P10451	OSTP	SPP1	(50)
Protein-lysine 6-oxidase	P28300	LYOX	LOX	(45)
Transforming growth factor-beta-induced protein ig-h3	Q15582	BGH3	TGFBI	(51)
Versican	P13611	CSPG2	VCAN	(52)
Vitronectin	P04004	VTNC	VTN	(28)
Elastic fibre associated molecules				
67-kD elastin-binding protein (EBP), spliced variant of β-galactosidase	–	–	–	(53, 54)
ADAMTS-like protein 3	P82987	ATL3	ADAMTSL3	(55)
ADAMTS-like protein 4	Q6UY14	ATL4	ADAMTSL4	(56)
ADAMTS-like protein 5	Q6ZMM2	ATL5	ADAMTSL5	(57)
Clusterin	P10909	CLUS	CLU	(58)
Endostatin - C-terminal fragment of collagen XVIII	–	–	–	(59)
Fibronectin	P02751	FINC	FN1	(60)
Integrin-binding sialoprotein	P21815	SIAL	IBSP	(61)
Matrix Gla protein	P08493	MGP	MGP	(62)
Protein-glutamine gamma-glutamyltransferase 2	P21980	TGM2	TGM2	(63)
SPARC (osteonectin)	P09486	SPRC	SPARC	(61)
Tenascin	P24821	TENA	TNC	(64)
Tenascin-X	P22105	TENX	TNXB	(65)

^a^ Accession numbers were retrieved from the Protein Database UniProtKB.

In blood vessels, elastin is present either in form of circumferentially aligned lamellae alternating in the media with syncytia of VSMC, or as a mesh of thin and loose fibres and sheets (i.e., valves) conferring the extensibility and the elastic recoil typical of these tissues. The number of lamellae decreases along the arterial tree, with larger elastic arteries having up to 60 layers in humans, whereas peripheral muscular arteries having less than 3 layers (67). In fact, the amount of elastin in blood vessels is highly dependent on their size and functional role related to the tensile cyclic forces acting radially and longitudinally on the vascular wall. The elastin content in large arteries is one and a half times that of collagen. Medium and small vessels, containing more VSMCs and less elastic tissue, are characterized by lower stretching capabilities. Veins have a similar structure, but exhibit a lower wall thickness compared to arteries and their elastic content is one third of that of collagen (68). The presence of the elastic component in blood vessels is fundamental for a proper function of the heart since elastin allows vessels to store a portion of the stroke volume with each systole and discharging that volume with diastole (Windkessel effect), thus decreasing the load on the heart, minimizing the systolic flow, and maximizing diastolic flow in the arterioles throughout the cardiac cycle (69).

In the valves, elastic fibres, although represented in a lower percentage (13%) compared to collagen (50%), are responsible for the extension of cusps beyond 50% and for the subsequent recoil. This finding indicates that mechanical properties are not dependent on the elastin content *per se*, but more likely to the organization and distribution of the fibres that are different in the *fibrosa* (the side of the valve that is more under compression and is more fibrotic with densely aligned collagen fibrils) and in the *ventricularis* (the side of the valve that is more under tension and is characterized by higher elasticity and radially oriented collagen fibrils) (70)*.*

### 2.1 Elastin synthesis and assembly

Elastin is a single gene-copy protein, although multiple isoforms can be produced by alternative splicing (71), and consistent differences in amino acid composition have been observed between vertebrate species and even between tissues in the same organism (72).

**Figure 2:**
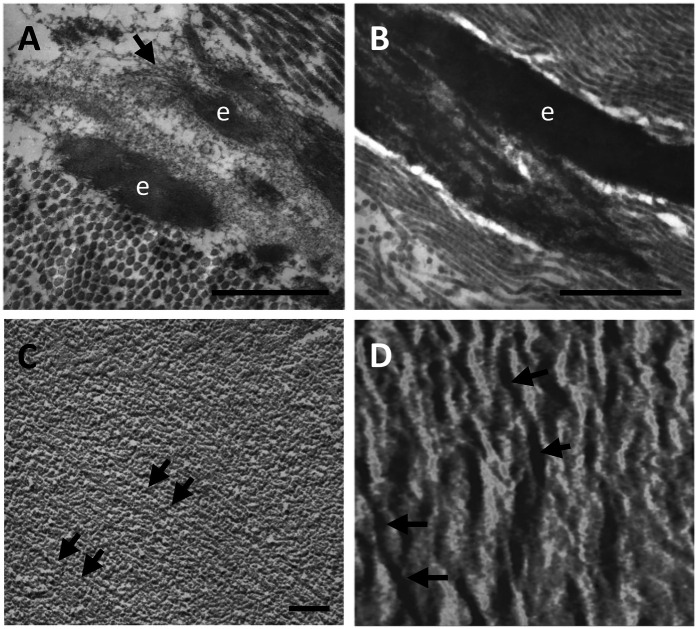
Elastic fibres (e) within the human aorta are shown, by transmission electron microscopy, as amorphous deposits onto a microfibrillar scaffold (arrow) **(A)** which over time progressively grow to form larger amorphous structures **(B)**. However, the elastic component is not completely amorphous. Indeed, an isolated elastic fibre from bovine nuchal ligament reveals parallel organized fibrils (arrows) both by freeze fractured transmission electron microscopy **(C)** and by atomic force microscopy **(D)**. Samples have been routinely processed for morphological analyses (19, 74) and figures are unpublished original images from the Authors' archive. Scale bar: 1 μm **(A,B)**, 0.1 μm **(C)**. Scanning range: 700 nm **(D)**.

Elastin is synthesized as a precursor of 60–70 kDa named tropoelastin (TE) that starts to be assembled in form of small globules within cells or at the cell surface before undergoing assembly and crosslinking by enzymes of the lysyl oxidase (LOX) family. TE is characterized by alternation of highly hydrophobic and hydrophilic domains, which are responsible for aggregation and formation of cross-links, respectively. The aggregation rate, the size, and other properties of the globules are modulated by several factors such as temperature, pH, ionic strength, concentration of individual TE monomers, and by the presence of elastin associated molecules. TE is characterized by high hydroxyproline content, although with differences according to tissues, being lower, for instance, in blood vessels compared to lung (1). Once synthesized, TE binds to an elastin binding protein that protects TE from self-aggregation and from proteolytic degradation during elastogenesis and acts as a chaperone to the cell membrane, where, together with two other membrane-bound components, it becomes part of the elastin-receptor complex (73). In the extracellular environment, an important step in elastin assembly is represented by the deposition of TE spherules onto a microfibrillar scaffold (Figure 2A) through the interaction with several molecules, most of them remaining entrapped within the elastic fibres (75). Mature elastic fibres are characterized by a typical amorphous structure although, by different morphological approaches, they exhibit a filamentous structure whose supramolecular assembly confers the elastic properties of the fibres (Figures 2B–D).

## 3 Calcification of the elastic component

**Figure 3:**
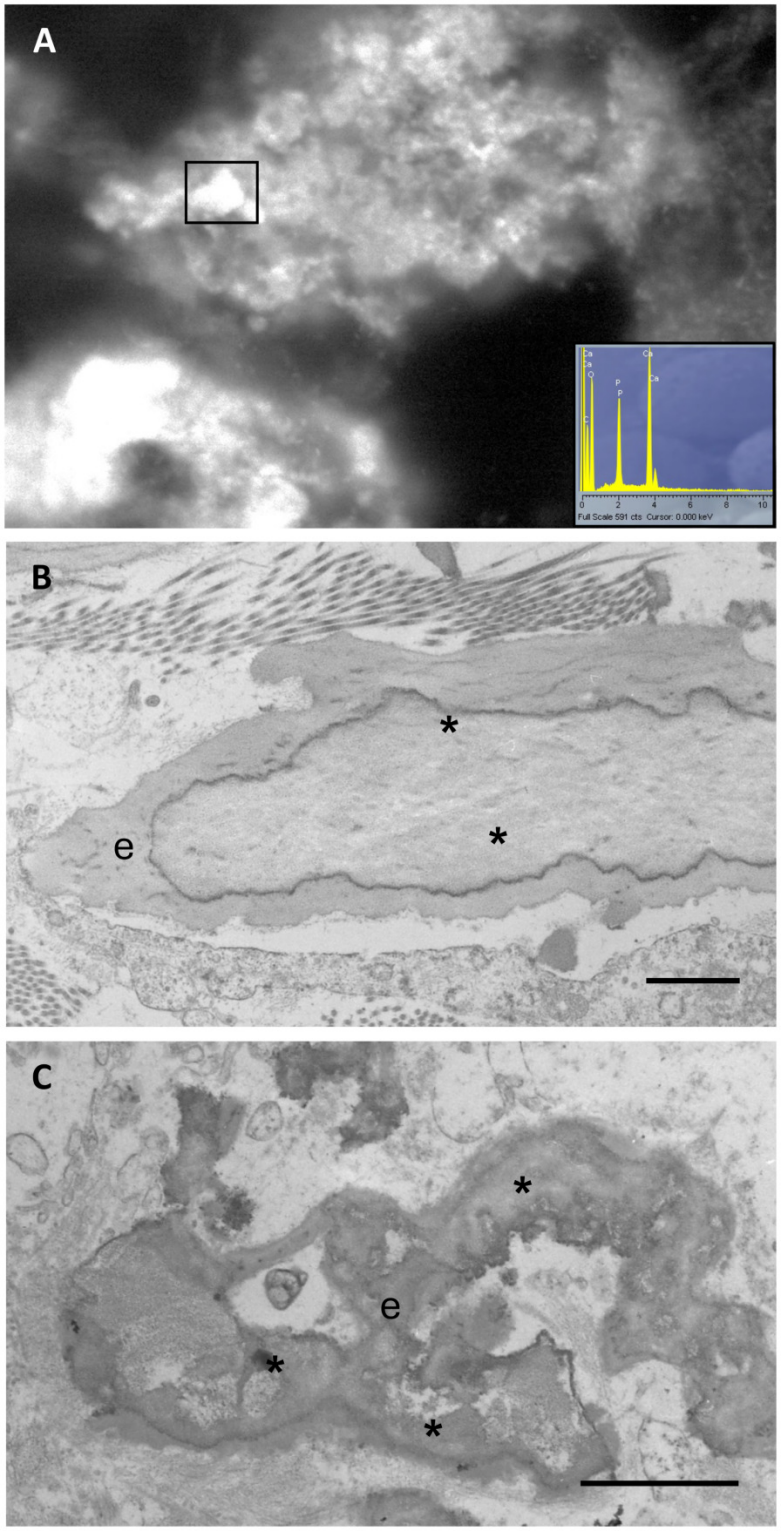
Calcified elastic fibres are observed by wet scanning electron microscopy and x-ray microanalysis (insert), demonstrating that bright mineral deposits (rectangle) consist of calcium and phosphate **(A)**. Calcification (*) in human arteries, observed by transmission electron microscopy, appears within the core of the elastic fibres (e) **(B)** or in form of deforming aggregates **(C)**. Samples have been routinely processed for morphological analyses (19, 21) and figures are unpublished original images from the Authors' archive. Scale bar: 1 μm **(B,C)**.

Elastin plays a crucial functional role in conferring tissue elasticity; however, it is also susceptible to calcium deposition and tissue stiffening (Figure 3).

Calcium phosphate deposits are usually formed through a multistep process starting from the development of amorphous calcium phosphate minerals [e.g., dicalcium phosphate dihydrate – DCPD - or brushite, CaHPO_4_⋅2(H_2_O)] followed by a transition to octacalcium phosphate [OCP, Ca_8_(HPO_4_)_2_(PO_4_)_4_⋅5H_2_O)] and apatite [Ca_10_(PO_4_)_6_(F,OH,Cl)_2_] or hydroxyapatite [HA, Ca_10_(PO_4_)_6_OH_2_] accompanied by progressive crystal growth (76). Crystal growth can be inhibited by chemisorption of pyrophosphate that, without attracting Ca as a counter ion, replaces Pi, a necessary counterpart of calcium in mineral deposits (77–79). It is interesting to note that polyphosphates, beside their inhibitory activity, can indirectly promote mineralization being a substrate of alkaline phosphatases that generates two phosphate ions capable of calcium binding (80).

The onset of calcification is not associated with surface precipitation of calcium phosphate, but it is more likely due to a complex biomineralization process that occurs within the bulk of the tissue (81). In addition, mineral–associated vesicles (MVs) from cells undergoing osteoblastic differentiation can nucleate and grow apatite crystals when bound to the elastin component (82, 83). However, evidence of elastin mineralization usually occurs after clinical manifestation or instrumental detection of mineral deposits in later stages of calcification, therefore the initial events triggering the process are still underexplored and the specific initial binding sites for calcium remain unclear.

Based on experimental models, two calcium binding sites have been proposed: neutral backbone carbonyl groups (C = O) and negative carboxyl groups (COO^−^), either as side groups of the aspartic acid and glutamic acid amino acids within elastin or as terminal groups of elastin-derived peptides. In the early 1970s, Urry proposed that backbone carbonyl groups act as calcium-binding sites, demonstrating that the binding occurred also in highly acid conditions (84, 85). Typically, elastin binds calcium through uncharged coordinating groups (i.e., neutral sites) progressively becoming positively charged and therefore attracting negatively charged ions such as phosphate, facilitating mineral deposition and further calcium ion binding (86).

Interestingly, coacervation and cross-linking, two key processes that in *in vivo* lead to elastic fibre formation, enhanced the exposure of backbone carbonyl groups, increasing their availability for calcium binding and subsequent mineralization (87). Indeed, Kaibara et al., investigating ion transport across a coacervate membrane composed of bovine neck ligamental α-elastin, found that calcium ion transport was mediated by specific and selective interactions with elastin neutral sites (88). In particular, the glycine-rich neutral sequences may act as nucleation sites for calcification also after elastin degradation (89).

However, other studies showed that also the carboxyl groups may participate in elastin calcification (90). Indeed, calcium ions bind to purified elastin in a manner dependent on both calcium concentration and pH, supporting a mechanism based on electrostatic interactions between Ca^2+^ and COO^−^ (91). Consistently, studies in non-calcified and calcified insoluble bovine elastin revealed that elastolysis caused a greater abundance of carboxyl groups in mineralized samples (92). Similar findings were also obtained using coacervated insoluble elastin fibrillar structures, which, even when they were only partially degraded by elastases, exhibited increased calcification due to the amount of negatively charged COO^−^ groups formed during elastin degradation (14). Therefore, the density of COO^−^ groups can influence not only the extent and the rate of elastin calcification but also the transformation of the CaP mineral phase (93).

Very recently, Lau and coworkers proposed that, during elastin calcification, backbone carbonyl groups represent the primary calcium binding sites (94), although the carboxyl groups may also participate (90). This hypothesis is supported by the high density of backbone carbonyl groups in elastin (95), the low turnover rate of elastin (3, 96), and the diminished electrostatic interactions of carboxyl groups in the aqueous environment (97, 98).

Overall, these data underline the complexity of the intermolecular interactions of elastin and indicate that the involvement of backbone carbonyl or of carboxyl groups may depend on the experimental conditions such as the use of elastin or of elastin-derived peptides, coacervated or not, degraded or non-degraded, and of the micro-environmental characteristics (e.g., pH, ion concentration and ion type).

**Figure 4:**
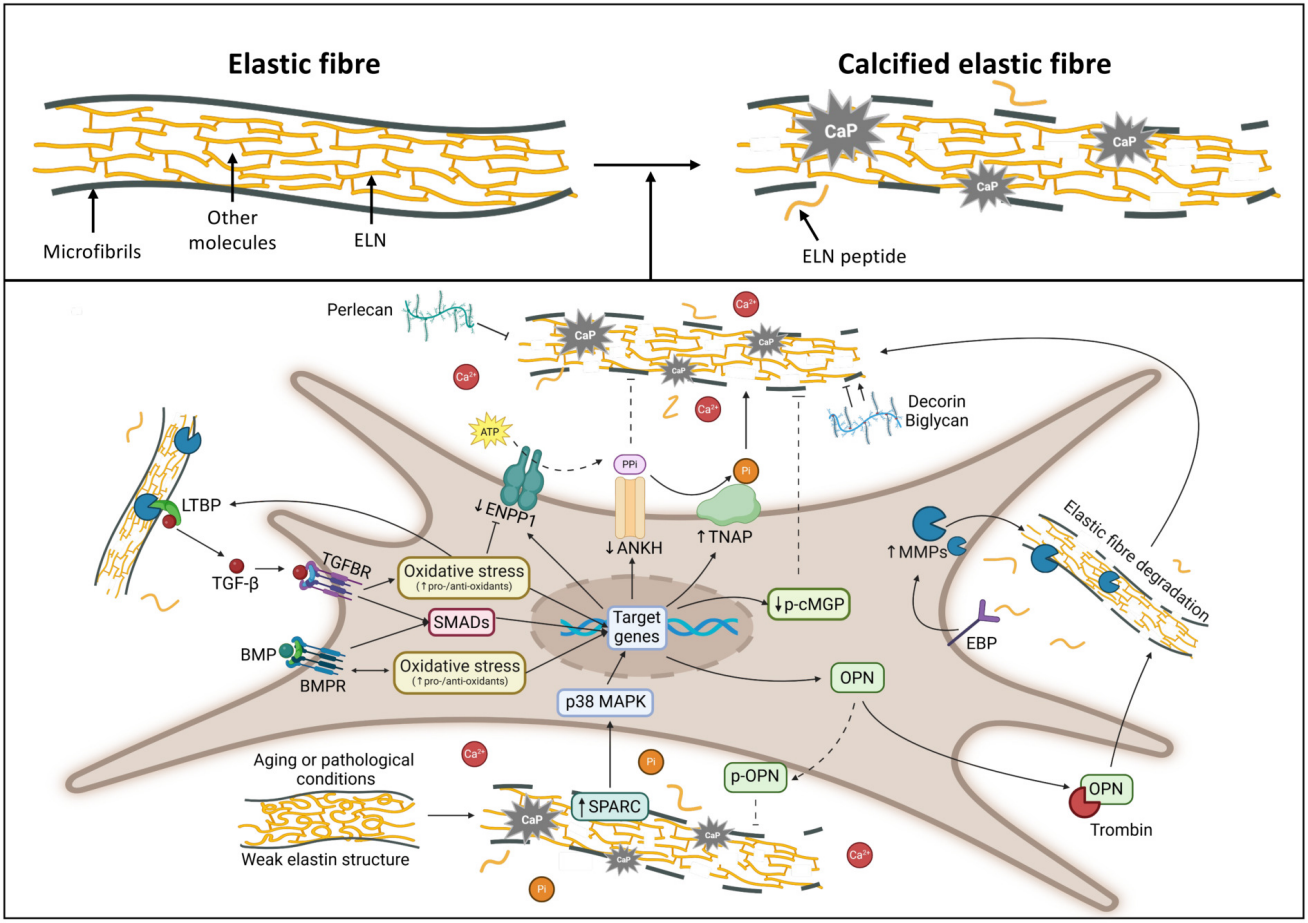
Drawing highlighting the major molecules and pathways involved in elastic fibre calcification. Created with https://www.BioRender.com. ANKH, progressive ankylosis protein homolog; BMPs, bone morphogenetic proteins; BMPRs, bone morphogenetic protein receptors; EBP, elastin binding protein; ELN, elastin; ENPP1, ectonucleotide pyrophosphatase/phosphodiesterase family member 1; LTBPs, latent transforming growth factor-beta binding proteins; MAPK, mitogen-activated protein kinase; p-cMGP, phosphorylated-carboxylated Matrix gla protein; MMPs, matrix metalloproteinases; OPN, osteopontin; p-OPN, phosphorylated osteopontin; Pi, inorganic phosphate; PPi, inorganic pyrophosphate; SMADs, small mother against decapentaplegic; SPARC, secreted protein acidic and cysteine rich; TGF-β, transforming growth factor beta; TGFBRs, transforming growth factor beta receptors; TNAP, tissue-nonspecific alkaline phosphatase.

Moreover, elastin calcification is associated not only with degradation but also with alterations in amino acid composition, which occur during aging (99) and/or in pathological conditions (e.g., atherosclerotic plaque progression) (100). These changes include an increase of polar amino acids, a decrease of non-polar amino acids, and a reduction of desmosine and isodesmosine content, leading to structural weakening of elastin (Figure 4), altered interactions with the numerous molecules exhibiting qualitative and quantitative changes with time and during disease, enhancing the susceptibility to an *in vivo* slowly occurring calcification process.

## 4 Elastic fibres components and associated molecules and their involvement in the calcification process

Elastic fibres are composed of several components but only for few of them it is known their involvement in the calcification process.

### 4.1 Fibrillins

As already mentioned, TE is progressively deposited onto a microfibrillar scaffold mainly composed of fibrillins (FBNs). FBNs (1–3) are cysteine-rich glycoproteins characterized by an extensible periodic beaded organization providing both structural and regulatory roles depending on the tissue and the developmental stage. In the vascular system, for instance, VSMC start to synthesize elastin, FBN1 and FBN2 from the mid gestational stage up to the neonatal life (101), although FBN2 seems to be required during development, whereas FBN1 appears mostly important in adults to maintain connective tissue homeostasis and integrity. *FBN1* variants are associated with the Marfan syndrome phenotype and, more generally, with cardiovascular, ocular and skeletal defects (102, 103), whereas mutations in *FBN2* affect the musculoskeletal system with only occasional cardiovascular signs (104). Interestingly, FBN3 is restricted to embryonic/foetal tissues and has been suggested to play a role in the female reproductive organs, although it is not expressed in animals such as mice and rats (105). In addition to their role as structural components of the microfibrillar scaffolds, FBNs influence the bioavailability of transforming growth factor-beta (TGF-β) and interact with cells through integrin-associated RGD sequences, as well as with cell surface heparan sulphate (HS) proteoglycans, fibronectin and collagen type VI (106).

Exome sequencing performed in beta-thalassemia patients with ectopic calcification, disclosing rare sequence variants in genes related to elastic fibre assembly and integrity, led to the hypothesis that altered fibrillins and collagen type VI molecules may affect the microfibrillar scaffold, thus making elastic fibres less resilient and more prone to hydroxyapatite deposition (107).

Since fibrillin microfibrils vary in structure, composition, distribution and possibly functional roles depending on the tissue, they can modify the susceptibility of elastic tissues to calcify, and can differently interact with molecules such as calreticulin and protein-disulfide isomerase (PDI) (108), known to be involved in the vitamin K-dependent carboxylation of matrix gla protein (MGP) (109, 110).

Moreover, fibrillins contain several calcium-binding EGF-like domains (111), thus suggesting that these residues may have a special impact in the calcification process (112), although it has been suggested that fibrillins may not support mineral deposition directly, but though its modulation of TGF-β (transforming growth factor-beta) and BMP (bone morphogenetic protein) signalling (113) (Figure 4).

Indeed, BMPs represent a large family (approximately 20 subfamily members) of secreted signalling molecules belonging to the TGF-β superfamily. Although firstly isolated from bones, thereafter it has been clearly demonstrated their ability to induce soft connective tissue calcification, especially for BMP-2, -4, -5, -6, -7 and -9. These BMPs, except BMP-7 that may have an inhibitory activity, have been localized in sites of vascular calcification (114). Both BMPs and TGF-β transduce signals through SMAD-dependent and -independent pathways (19) requiring complex regulatory mechanisms (e.g., latency control in the matrix, extracellular antagonists, ubiquitination and phosphorylation in the cytoplasm, nucleus-cytoplasm transportation, and transcriptional co-regulation in the nuclei) (115). SMAD-signalling pathways (Figure 4) are crucial for the activation of several pro-osteogenic genes in the context of elastin metabolism and elastocalcinosis (116).

### 4.2 Glycosaminoglycans and proteoglycans

The interactions between elastin and GAGs have been demonstrated for decades (117, 118). GAGs are negatively charged polysaccharides, which can be either sulphated (heparan sulphate, HS; chondroitin sulphate, CS; keratan sulphate, KS; dermatan sulphate, DS) or non-sulphated (hyaluronic acid), and are covalently attached to a core protein to form a huge variety of PGs with different tissue distribution (119). They are widely present in the ECM, but also in the pericellular areas, on the cellular surface and in intracellular secretory granules (120). According to their characteristics, PGs modulate how cells respond to the ECM and *vice versa*. Indeed, they contribute to tissue hydration, interact with growth factors, cell surface receptors and ECM molecules regulating not only protein assembly and supramolecular organization, but also modulating cell adhesion, proliferation, migration, differentiation, apoptosis and cell signalling (120). In the extracellular environment, GAGs interact with TE and with FBNs promoting the assembly of TE and of the microfibrillar scaffold (121–123). HS, in particular, although it does not seem to influence elastin gene expression and/or the elastin synthesis, promotes aggregation and assembly of isolated elastin molecules and of elastin peptides and increases the expression of fibulin 5 in cell cultures also from aged donors (124, 125).

GAGs and PGs in vascular disease have been largely investigated due to their role in maintaining vascular architecture and their increases in pathological conditions, thus altering the proportion of other components (e.g., collagen and elastin) as well as their reciprocal interactions with cells (126). It has been proposed that GAGs accumulation in the vessel wall may serve as an adaptive compensatory response to increased mechanical stress and inflammation, since GAGs, attracting water molecules, cause a tissue swelling which may help absorbing mechanical stress as observed in aged and/or in diseased vessels (127).

The importance of GAGs and PGs in the mineralization process has been extensively investigated due to their hydrodynamic properties (depending on molecular weight, molecular size and viscosity) and high charge density interfering with the diffusion of calcium and phosphate ions (128–130). It has been proposed that negatively-charged groups in PGs interact with active growth sites on the HA nuclei to reduce apatite growing or that, depending on their size, may shield the active sites on the HA seed crystals from the availability of calcium phosphate ions (131). Consistently, it has been recently highlighted that pretreatment of bioprosthetic heart valves with a “HPA/NT/HRP” enzyme-oxidative-polymerization strategy exerts anti-calcification properties by improving GAGs stability, thus prolonging the efficiency of these valves substitutes (132).

However, evidence has been also provided for a possible role of some PGs as inducers of calcification in blood vessels (133). For instance, perlecan, a HS-PG, interacts with elastin, FBN 1 (134) collagen type VI and with several growth factors and signalling molecules (e.g., fibroblast growth factors, platelet-derived growth factor, vascular endothelial growth factor and BMPs) (135). It has multiple biological functions, promoting angiogenesis, tissue development and ECM stabilization playing a role as a blood shear-flow endothelial sensor that regulates blood volume and pressure (135) and consequently intercellular crosstalk. Reduced expression of perlecan was demonstrated in calcified atherosclerotic arteries (Figure 4), in contrast with the increased production of CS- and DS-PGs (136). The calcification inhibitory effect of perlecan can be related to the high presence of HS that exerts an important role in elastin coacervation and fibre assembly (125).

On the contrary, aggrecan, a CS-/KS-PG also known as cartilage-specific proteoglycan core protein, is up-regulated in the atherosclerotic plaques, where, by favouring lipoprotein accumulation, acts as an inducer of chronic inflammation promoting VSMC trans-differentiation (137).

Interestingly, decorin (a secreted CS-/DS-PG), and biglycan (a small leucine-rich repeat PG) act both as promoters and inhibitors in the mineralization process (138) (Figure 4). This apparent discrepancy is due to differences in the environmental conditions. In a cell-free model, decorin and biglycan bind to hydroxyl apatite crystals reducing hydroxyapatite crystal growth. By contrast, in a more complex scenario, the presence of growth factors, and of specific electrostatic interactions of PGs with LDL (low density lipoproteins), both in blood vessels and in valve leaflets (139), can favour calcification of the elastic component enhancing the interactions of elastin with fatty acids and lipophilic compounds (140). Indeed, elastin is known to have high affinity for lipids and calcium and to be more susceptible to elastolytic activities if fibres are altered by the enriched presence of lipids (141, 142).

### 4.3 Tenascin

Tenascin-C is a glycoprotein regulated by mechanical and tensile stress during development and in adult life, and colocalizes with elastin only in mineralized areas. Indeed, calcified elastin displayed a time-dependent pattern of tenascin-C, MMP-12 (matrix metalloproteinase-12), and alkaline phosphatase expression (64, 143). Similarly, tenascin-X, a large glycoprotein of the ECM which harbors binding site for collagens, decorin and elastin, is expressed in the vascular wall and in particular in association with the elastic lamellae (65). It has been implicated in the occurrence of a subset of Ehlers Danlos Syndrome (144) where, beyond collagen abnormalities, also irregular, fragmented and branching elastic fibres can be observed possibly due to altered interactions with collagen type VI (145). It is also worth mentioning that expression of tenascin-X is significantly reduced in calcific aortic valves (146).

### 4.4 Matrix Gla protein

Matrix Gla Protein (MGP) is expressed by VSMC in the intima and the media of vessels and undergoes post-translational modifications important for its activation and function (i.e., vitamin K-dependent carboxylation of five *γ*-glutamic acid residues and serine phosphorylation). For instance, MGP-derived peptides containing both post-translational modifications, added into calcium and phosphate solutions, significantly influence the nucleation and growth of HA in contrast to unmodified peptides. This model allowed to demonstrate that phosphorylation is more effective than carboxylation in modulating mineralization (147). Reduced expression of MGP has been associated with elastin calcification in Monckeberg's sclerosis (148), in *Pseudoxanthoma elasticum* (109), in thalassemia patients (149), in Singleton-Merten syndrome (150) and in knockout mice (151), demonstrating that p-cMGP (phosphorylated-carboxylated MGP) acts as an inhibitor of calcification (152) (Figure 4). It has been hypothesized that MGP can inhibit trans-differentiation of VSMCs and/or the formation of matrix vesicles (153). In the calcification process matrix vesicles have a functional role not only as a reservoir of HA and matrix components acting as intercellular signalling modules, but also as a source of MMP-2 that, by closely interacting with elastic fibres, favours their degradation and subsequent calcification (83). Mutations of the *MGP* gene cause Keutel syndrome characterized by a complex phenotype, including extensive arterial calcification, as clearly shown in *Mgp^−/−^* mice that die before 2 months of age (154, 155), exhibiting pronounced vascular calcification (151). The observation that MGP is colocalized with elastin (62) led to the hypothesis that the elastin content may be a key regulator of arterial calcification in *Mgp*^–/–^ mice. Indeed, elastin haploinsufficiency can decrease arterial mineral accumulation (152).

### 4.5 Osteonectin

Osteonectin, also known as SPARC (secreted protein acidic and rich in cysteine) or BM40 (basement membrane 40), is one of the most abundant non-collagenous proteins expressed in mineralized tissues with high affinity for HA. It is localized in calcified areas on stromal cells and within elastic fibres and occasionally in association with lipids (61, 143). It has been suggested that osteonectin regulates the extracellular matrix mineralization through the p38 MAPK signal transmission pathway (Figure 4) that has a broad regulatory effect on alkaline phosphatase activity, on the response to osteogenic signals driven by BMP-2, TGF-β, parathyroid hormone, Wnt proteins, mechanical stimuli and pro-osteogenic transcription factors such as Runx2 (156).

### 4.6 Osteopontin

Osteopontin (OPN) is a highly phosphorylated sialoprotein found in mineralized extracellular matrices where it mediates HA binding through polyaspartic acid sequence and sites of Ser/Thr phosphorylation (157), although, more recently, it has been demonstrated to play multiple modulatory roles including vascular remodelling (158), supporting the observations that it is present in normal elastic fibres (61). While osteopontin, in its full-length phosphorylated form, has been shown to inhibit calcium phosphate production (Figure 4), other sialoproteins, such as bone sialoprotein, can act as a nucleator of calcium phosphate formation. This behaviour can be related to the type of interactions with mineral crystal nuclei depending on the primary structures of the sialoproteins and to the extent to which they are phosphorylated. More specifically, polyglutamate-containing bone sialoprotein acts as a nucleator, while the polyaspartate-containing osteopontin inhibits calcium phosphate formation and growth (159). Although osteopontin is relatively protease-resistant, it has been shown that it can be hydrolysed by thrombin (Figure 4) exposing a cryptic α_4_β_1_/α_9_β_1_ integrin-binding motif (SVVYGLR), thus exerting pro-inflammatory, pro-angiogenic and pro-osteogenic effects favouring aortic MMP-9 activation (160).

## 5 Elastin degradation and the calcification process

Due to its almost absent turnover, elastin is characterized by high resistance and durability. However, there are several elastolytic enzymes, belonging to the class of metallo-, cysteine- and serine-endopeptidases, that can degrade elastic fibres (i.e., TE, elastin and FBNs) (161) (Figure 4), lowering their functions, and at the same time releasing bioactive peptides called elastokines (3). These fragments, depending on their amino acid sequence, can interact with different types of receptors inducing a variety of biological activities (e.g., angiogenesis, cell proliferation, vasorelaxation), which may lead to the development of age-related morpho-functional alterations and/or to the progression of pathological conditions (162, 163). Overproduction of elastolytic enzymes and/or a decrease of some of their inhibitors are frequently associated with inflammatory conditions where the release of elastin fragments with chemotactic properties for monocyte contributes to structural deterioration (e.g., atherosclerosis), enlargement and eventual rupture of large vessels (e.g., aortic aneurysms) and promote osteogenic differentiation and subsequent calcification (1, 23, 164, 165). Metallopeptidases activity is regulated either at the mRNA level or by proenzyme activation depending on substrate affinity/specificity, on the presence of specific inhibitors or on binding to other matrix molecules such as GAGs, PGs and other glycoproteins (e.g., tenascin-C) (143, 166, 167).

In particular, MMP-1, MMP-2, MMP-3, MMP-7, MMP-9 and MMP-12 have been demonstrated to bind to and to digest insoluble elastin (168, 169), their activation being associated with cardiac valve diseases, atherosclerosis, aortic aneurysms, and restenosis (7). Indeed, enhanced elastin calcification is directly associated with increased MMP expression (i.e., *MMP-2* and *-9*), and also with up-regulated expression of osteogenic genes (e.g., *ALP*) (169, 170) (Figure 4).

Similarly, it has been reported, both *in vitro* and *in vivo,* that the elastolytic activity of cathepsin K, S and V may have a role in the progression of atherosclerosis as well as in vascular and valve calcification (89, 171). Since cathepsin activation can be regulated by interactions with GAGs and/or PGs (172), variations of tissue composition in terms of GAGs/PGs, and possibly other matrix components, may contribute to the different susceptibility to ectopic calcification of tissues and/or of areas within the same tissue (173).

## 6 Functional consequences of calcification in the cardiovascular system

Elastic fibres are present in all soft connective tissue and, with age, dermal, cardiovascular, and pulmonary tissues become less able to recoil, leading to the hypothesis that age-related failure of elastic fibres may dictate the apparent 100–120-year limit on human life expectancy (174). Nevertheless, the major clinical relevance of elastin calcification is observed in the cardiovascular system causing stiffening of arterial walls, higher values of systolic pressure, and increased incidence of cardiovascular events (175). Similarly, aortic valve calcification is the most common valve disease in the developed countries, frequently requiring heart surgery and/or transcatheter valve replacement since prolonged valve calcification may alter the heart's structure (e.g., enlargement or thickening of the heart walls) affecting cardiac function (176). Aortic valves leaflets are susceptible to risk factors similar to those of atherosclerosis, moreover they are constantly subjected to mechanical stress but if these stimuli exceed the physiological range or if the capability to respond to mechanical stress is insufficient, valves undergo calcification with increased expression of osteogenic markers (e.g., BMP2 and RUNX2), changes in the behaviour of valve interstitial cells and altered composition/organization of the extracellular components (177, 178).

Vascular calcification is a typical complication occurring in patients with diabetes, atherosclerosis, chronic kidney disease, hypertension, thus causing a progressive stiffening of blood vessels with altered blood flow and altered mechanical signals provided to endothelial and smooth muscle cells and their crosstalk (179).

Indeed, blood vessels are typically exposed to the pulsatile pressure formed by heart contraction, however, their morphological structure and organization exert different mechanical and hemodynamic properties due to blood flow velocity gradient on the surface of the vessel wall and blood viscosity (180). Indeed, the wall shear stress (WSS) acting on the vessel wall ranges from 1 dyne/cm^2^ in the venous endothelium to 40 dyne/cm^2^ in the calcified arteries (181). Within this context, the ratio between collagen and elastin and tissue elasticity are crucial for maintaining a dynamic balance between growth and remodelling under a certain range of blood pressure and consequently modulating the organization and alignment of endothelial cells. Compared to arteries, despite the significantly lower WSS, veins exhibit lower levels of calcification, possibly due to less organized layers of elastic lamellae and smooth muscle cells that are also characterized by lower osteogenic trans differentiation. Furthermore, the lower venous pressure and the presence of venous valves, maintaining unidirectional blood flow and preventing blood reflux, can play a role in preventing calcification (181).

Within arteries, calcifications occur in the different layers of the wall depending on the disease context. For instance, intimal calcification, that is mainly involved in atherosclerosis, begins with microcalcifications (less than 20 μm in size), originating from the lipid pool and the necrotic core, and then progressively developing into sheet, nodular or plaque calcifications representing predictors of unstable plaque at the risk of rupture, whereas extensive calcification is associated with plaque stability. On the other side, medial calcification is a chronic systemic vascular disease commonly seen in diabetes, chronic kidney disease and is characterized by spread calcium phosphate deposits, leading to decreased compliance of the whole vessel wall and to increased incidence of cardiovascular complications. Therefore, medial calcification is considered a strong and independent risk factor for cardiovascular death and predictor of risk of future coronary events in patients with diabetes (182).

Indeed, calcification in peripheral artery disease (PAD) has multiple consequences accelerating pulse wave velocity from heartbeats, impairing microcirculation and reducing the artery's capacity to adjust blood flow according to physiological demands thus impairing tissue perfusion (183). In the lower extremity arteries, calcification poses additional challenges due to the deformations of blood vessels and of blood flow during limb flexion and eventually to microfractures of mineralized plates causing the release of cellular debris, calcium crystals, matrix peptides and lipid residues (184).

In agreement with these finding coronary artery calcium content is regarded as an arterial manifestation with prognostic value, and extra coronary calcification (e.g., calcification of the aorta or mitral valve) is also independently associated with cardiovascular disease (185). The ARIC (Atherosclerosis Risk in Communities) Study reported that Pulse Wave Velocity (PWV) is strongly associated with descending aorta calcification and that among PWV measures, cfPWV (carotid femoral PWV) was most strongly associated with vascular and valvular calcification, followed by hfPWV (heart-femoral PWV) and haPWV (heart-ankle PWV). Interestingly, faPWV (femoral-ankle PWV) was inversely associated with vascular calcification (186).

Interestingly, microcalcifications have been described also in thoracic aneurysms of various aetiologies and these events are specifically associated with elastin fragmentation, vascular smooth muscle cell phenotypic switching, and increased aortic wall rupture risk on biomechanical testing. Higher microcalcifications have been observed associated with mild and moderate histopathologic thoracic aortic aneurysm severity, whereas severe pathological disease was associated with low levels of microcalcification, suggesting a nonlinear pathobiological course. While elastin fragmentation is known to have an augmenting effect on microcalcification, however total elastin destruction or loss seen in the end-stage disease have the opposite effect. This finding must be taken into account when assessing histological severity by microcalcification (187).

## 7 Models of elastin calcification

Given the complexity of the ectopic calcification process and the difference in the morpho-functional characteristics within the cardiovascular system, experimental models are crucial for understanding mechanisms, testing hypotheses, and developing new treatments. These models range from simple acellular systems to *in vitro* models of increased complexity and to animal models either treated with pro-osteogenic stimuli of genetically manipulated to better dissect the role of specific molecules and/or pathways. Each model has advantages and disadvantages (Table 2), although they are always capable to provide insightful information for translational perspectives.

**Table 2:** Strengths and limits of model systems.

Main features	Acellular model	2D cell culture	3D cell culture (spheroid model)	3D cell culture (organoid model)	Invertebrate animal model	Vertebrate animal model
Easy to be established	Yes	yes	Yes	No	Yes	No
Easy to maintain for long time	Yes	Yes	Challenging	Challenging	Yes	Yes
Specialized facilities and expertise	No	No	Yes	Yes	Yes	Yes
Relative cost	Low	Low	High	Higher	Low	Higher
*In vivo* similarities of cell shape and differentiation	No	No	Yes	Yes	Yes	Yes
Cell-cell interactions	No	Limited	Yes	Yes	Yes	Yes
Cell-matrix interaction	No	Limited	Limited	Yes	Yes	Yes
Genetic manipulation	No	Yes	Yes	Yes	Yes	Yes
Physiological complexity	No	No	No	Limited	Yes	Yes
Recapitulation of human physiology/pathology	No	No	No	Limited	Limited	Partly
Ethics Committee approval	No	Depending on the source and type of cell line	Depending on the source and type of cell line	Yes	No	Yes

### 7.1 *In vitro* models

Elastin synthesis can be induced *in vitro* by cytokines and growth factors (e.g., interleukin 10, TGF-β, insulin-like growth factor) (188, 189) and/or matrix molecules (e.g., hyaluronan) (190), although elastic fibre development is almost negligible, thus limiting the study of elastogenesis *in vitro* (191). In fact, to be assembled, elastic fibres require the presence of a microfibrillar scaffold, as demonstrated in immortalized ciliary body pigmented epithelial cells (192) and in cardiovascular tissue equivalents (191); nevertheless, to our knowledge, these models were not used to investigate calcification of the elastin component.

Therefore, most of *in vitro* cell culture models, even using co-culture with endothelial and VSMC (193), investigate the calcification potential of cells, upon treatment with culture media supplemented with phosphate or with ascorbate, dexamethasone and beta-glycerophosphate (4), looking at mineral deposition of the overlaying extracellular matrix, but not specifically on elastic fibres.

To address the challenges associated with the *in vitro* development of elastic fibres, alternative models employing formed fibrils or elastin peptides have been proposed. It is well known that calcification entails the formation of different mineral crystal phases, which vary based on the pathological context and the physicochemical characteristics of the extracellular environment (194–197). Therefore, these *in vitro* systems provide valuable tools for investigating the nature and progression of mineral deposition and for assessing compounds with potential anti-mineralization properties.

For instance, cross-linked elastin-like polypeptide (ELP) membranes are employed to mimic medial arterial calcification processes (198, 199). In particular, Gourgas and collaborators developed synthetic elastin-like polypeptide membranes (ELP_3_), that recapitulate key motifs found in human tropoelastin (198, 200) and, when immersed in simulated body fluid (SBF), they represent a suitable substrate for mineral nucleation and growth (201) depending on SBF ionic strength and incubation time (200). This model further highlighted that amorphous calcium phosphate (ACP) and OCP act as transient precursor phases during mineralization, in agreement with observations in the *Mgp^−/−^* mice, where both ACP and OCP were detected in the medial layer of calcified arteries prior to transformation into more stable apatite forms (201). The model of the ELP_3_ membranes has also been used to assess the effect of calcification inhibitors such as m3pS, a synthetic peptide corresponding to the N-terminal region of MGP containing three phosphoserine residues (199). Interestingly, ELP_3_ membranes, incubated for several days in SBF with or without m3pS, showed that, in the absence of the peptide, both OCP and carbonated hydroxyapatite were detected, whereas m3pS-treated samples showed only trace amounts of DCPD and minimal HA formation. These results suggested that the presence of m3pS altered the mineral maturation and that serine phosphorylation in MGP played a direct role in preventing mineral phase maturation in *in vitro* elastin mineralization process (199).

It is noteworthy that ELPs, due their inverse temperature transition properties (i.e., a phenomenon where a substance becomes more ordered, or folds, as the temperature increases) and biocompatibilities, can be efficiently used for drug delivery, as demonstrated *in vitro* for the prolonged release of BMPs (up to 14 days) and their enhanced mineralization capabilities (202, 203).

Additionally, intrinsically disordered proteins (IDPs) have recently been suggested to represent an interesting model also for the calcification process (204). IDPs are proteins that, lacking a stable and fixed three-dimensional structure, exist in a dynamic ensemble of conformations, allowing to interact with multiple partners regulating cellular processes like signalling, gene regulation, and molecular recognition (205). Therefore, IDPs can establish intermolecular interactions at the protein–mineral interface, thereby modulating the nucleation and crystal growth (206). For instance, it has been observed that amelogenin, a highly conserved IDP, influences enamel biomineralization by transitioning from a disordered random coils structure to an ordered β-sheet conformation upon binding to forming crystals (207). In the light of these data, it has been recently explored the mineralization potential of IDP elastin-like recombinamers (ELRs) (208) that, due to their biocompatibility and biodegradability, are well-suited for biomimetic applications (209). These ELRs are recombinant polypeptides inspired by the repetitive elastin sequence Val-Pro-Gly-*X*-Gly (VPGXG), where *X* represents any amino acid except proline, typically found in the intrinsically disordered regions of tropoelastin which are characterized by a phase transition from a soluble unidimensional disordered chain of amino acids into a stable coacervate capable of complex intermolecular interactions (210). In particular, ELRs, composed by two distinct tropoelastin motifs, a hydrophobic sequence (VPGIG) and a positively charged segment (VPGKG where K serves as cross-linking site), when incubated in supersaturated mineralizing solution, induced the formation of ELR spherulites functioning as nucleation and templating sites for Ca-P crystallization not only on the surface but also throughout the internal structure of assembled ELRs (208).

Similarly, isolated insoluble bovine elastin coacervated *in vitro* to form fibrillar structures can be used in a reductionist approach to test the mineralization potential and/or to investigate the regulatory role of standard or of calcifying medium, with or without addition of pro- or anti-osteogenic molecules. For instance, chemical and enzymatic fragmentation of the fibrils markedly increased HA deposition. Furthermore, variations in the composition of calcifying medium, such as the use of DMEM enriched with HPO_4_^2−^ compared to HEPES buffer solution supplemented with both Ca^2+^ and HPO_4_^2−^, modulate the extent of elastin calcification (14). The same *in vitro* model was used to test the mineralization inhibitory potential of GAGs. In particular, elastin fibrils were coacervated in the presence or absence of different concentration of HS, enzymatically digested and subsequently incubated in a calcifying medium. The results demonstrated an inverse correlation between HS concentration and the amount of calcium deposition on elastin fibrils (169). These data support the hypothesis that elastin degradation precedes calcification and underscore the importance of ionic environment as well as the type and the amount of extracellular matrix associated molecules, such as GAGs, in modulating mineral deposition on elastin matrices.

In the last years, nanomaterials, and in particular nanoparticles, have raised a huge interest for their physico-chemical properties, lower toxicity and biocompatibility (211). In the field of cardiovascular calcification, innovative and interesting models are represented by a tissue culture set-up using porcine aortic valve leaflets (212) or by murine aortic ring sections (213) that can be stimulated to calcify *ex vivo*. Samples treated for one week with human serum albumin-based nanoparticles conjugated with the chelator diethylenetriaminepentaacetate (DTPA) and with anti-elastin antibodies to specifically target calcified elastin exhibited reduction of calcium content (38%–46%) either in terms of progression of calcification or of reduction of existing crystals. The main advantage of the valve leaflet model is that valve interstitial cells are preserved maintaining interactions with the extracellular matrix and endothelial cells. However, the disadvantage is the transferability to humans because of the differences in the microstructure of porcine and human valves (212).

### 7.2 *In vivo* models

*In vivo* models are typically comprised by several mice knockout for specific molecules either related to elastic fibres or to the calcification process (155, 214, 215) or a combination of double knockout either in the homozygous or heterozygous condition depending if the genotype is life-compatible (Table 3). However, only in few cases a clear description of the calcification of the elastic component has been clearly reported.

**Table 3:** Mice models deficient for elastic fibres components and/or for promoters/inhibitors of calcification and exhibiting elastic fibres mineralization.

Mouse models	Gene	Role/disease model
*Eln^+/−^*	Elastin (*Eln*)	Structural component of elastic fibres. Animals have reduced susceptibility to vascular calcification (152).
*Fbn-1^+^/mgR*	Fibrillin1 (*Fbn*)	Major component of elastin microfibrillar scaffold. Reduced expression of the monomer (15% of normal allele) is associated with focal calcification of intact elastic laminae (216).
*Mgp^−/−^*	Matrix gla protein (*Mgp*)	Vitamin K-dependent protein with anti-mineralization properties. The amount of deposited minerals in *Mgp^−/−^* arteries scales with elastin amounts, decreasing from the thoracic to the abdominal aorta (152, 217).
*Mgp^−/−^Eln^+/−^*	Matrix gla protein (*Mgp*) and elastin (*Eln*)	Animals have markedly reduced arterial mineral deposition compared with *Mgp^−/−^*mice indicating that also the elastin content is a critical determinant of arterial medial calcification (152).
*MMP3* flox^+/+^	Matrix Metalloproteases 3 (*MMP3*)	Matrix metalloproteinase with elastolytic activity. Animals exhibit suppressed phosphate-induced SMC osteogenic transformation and medial artery calcification (218).
*Spp1^−/−^*	Osteopontin (*Spp1* also named *Opn*)	Inhibitor of mineralization present also within elastic fibres (219, 220).
*Phospho1^−/−^*	Phosphoethanolamine/Phosphocholine Phosphatase 1 (*Phospho1*)	Participates in the initiation of hydroxyapatite (HA) deposition inside MVs by scavenging P_i_ from phosphoethanolamine and phosphocholine (221).
*Phospho1^−/−^Spp1^−/−^*	Phosphoethanolamine/Phosphocholine Phosphatase 1 (Phospho1) and Osteopontin (*Spp1*)	Ablating *Spp1* function prevents the development of the skeletal phenotype of *Phospho1^−/−^* mice, establishing a primary role for OPN, rather than PP_i_, in the pathophysiology of the *Phospho1^−/−^* defects (221).
*Enpp1^−/−^*	Ectonucleotide pyrophosphatase/phosphodiesterase (*Enpp1*)	The enzyme generates PPi, a strong inhibitor of ectopic mineralization. Animals exhibit abnormalities almost identical to those present in the tiptoe walking (ttw/ttw) mice such as altered bone mineralization and arterial calcification. Used model for Generalized Arterial Calcification in Infants (GACI) (222).
*Enpp1^asj/asj^*	Ectonucleotide pyrophosphatase/phosphodiesterase (*Enpp1*)	The presence of an inactivating mutation significantly reduces ENPP1 function. Animals have a median lifespan of 58 days and histologic examinations reveal calcification in hearts and aorta (223).
Alpl^−/−^	Alkaline phosphatase (*Alpl* also named *Akp2)*	The enzyme promotes mineralization catalysing the hydrolysis of the inhibitor PPi while concomitantly increasing the levels of Pi. It is involved in medial artery calcification. Animals are characterized by elevated levels of OPN whose amount correlates with the severity of hypophosphatasia (221, 224).
*Ank^−/−^*	Progressive ankylosis protein homolog (*Ank* also named *Ankh*)	A multipass transmembrane protein involved in the transport of the inhibitor PP_i._ (224).

Since aging is frequently associated with an increased susceptibility to pro-calcifying factors (225), the Klotho-deficient mice, a well-known model of premature aging, developing also arterial calcification and elastin fragmentation, is used to investigate anti-calcifying treatments and to further elucidate the signalling pathways connecting aging and elastocalcinosis (226, 227). Indeed, Klotho is an aging-suppressor gene, but it acts also as a co-receptor for fibroblast growth factor-23 (FGF23) establishing the FGF23-Klotho endocrine system required for the maintenance of calcium-phosphate homeostasis (228). Klotho deficiency is correlated, for instance, to high levels of Bmps, Runx2 (an osteoblast transcription factor), phosphorylation of Smad1/5/8 and Smad2/3, osteoprotegerin, osteopontin and alkaline phosphatase (227, 229). Within this context, treatment with acetazolamide, an inhibitor of carbonic anhydrase used to promote diuresis thus lowering hypertension, was demonstrated to reverse the aging phenotype of the Klotho deficient mouse significantly reducing vascular calcification and osteogenic trans-differentiation (229).

In addition, an interesting model is represented by *Abcc6^−/−^* mouse resembling the genetic disease *Pseudoxanthoma elasticum*, where progressive calcification of elastic fibres has been associated to mutations in a gene encoding for a liver transmembrane transporter (230, 231), whose pathogenic role has not yet completely understood, further supporting the complexity of mechanisms leading to ectopic calcification. Therefore, this animal model has been frequently used to support and/or to validate results from studies in cell culture systems. It is the case, for instance, of the role of oxidative stress (110, 232, 233), vitamin K-dependent carboxylation of MGP (109, 110, 234, 235), alkaline phosphatase and pyrophosphate metabolism (236–239). Most importantly, using the *Abcc6^−/−^* model, in which the ectopic calcifications can be observed starting from 5 weeks of age, it was possible to highlight which are the alterations due to the gene deletion *per se* or the consequences of the calcified environment (240). Moreover, in the *Abcc6^−/−^* model, it has been demonstrated that progressive ankylosis protein homolog (Ank) and OPN expression are downregulated already before mineral deposition, whereas increased intracellular O_2_^−^ levels, elevated TNAP activity, and Bmp2 upregulation are observed after the occurrence of calcification (240).

Invertebrate animal model (e.g., *Saccharomyces cerevisiae*, *Drosophila melanogaster*, *Danio rerio*) are widely used by researchers to study specific biological processes, since they have genetic characteristics similar to humans, are easy to maintain and to be reproduced in a laboratory setting, and easy to generate mutants suitable to explore certain traits or diseases.

For instance, in the cardiovascular context, zebrafish represents an interesting invertebrate animal model for its ability to produce elastic fibres. Although zebrafish expresses the a*bcc6a* gene and exhibit morphogenetic changes upon the insertion of specific sequence variants (241), the resulting phenotype only partially mimics that of PXE, as ectopic mineralization is rarely observed in zebrafish mutants (242). In fact, in contrast to the limited expression of the gene in the liver of mammals, a*bcc6a* is expressed in the eyes, heart, and intestines and not in the liver of young adult zebrafish (241). By contrast, the zebrafish expressing *enpp1* mutants (i.e., model of the Generalized Arterial Calcification of Infancy) exhibit calcification of cartilage, skin, and cardiovascular system, such as the bulbus arteriosus (243).

A promising *in vivo* model is represented by subcutaneous implantation of glutaraldehyde-crosslinked vascular tissues into young rats, allowing to develop HA deposition onto elastic laminae (244). Results underline the importance of the environment in the calcification of the elastic component as it occurs in young patients undergoing valves replacement. In fact, children have higher serum phosphate, osteocalcin, parathormone and vitamin D compared to adults and consequently young patients have higher incidence of complications due to mineral deposition (245). The subdermal implant can be used to investigate the modulatory effects of exogenous molecules, such as Al^3+^ ions that were demonstrated to bind irreversibly to elastin, inducing conformational changes characterized by a reduction of the extent of β sheet structures and an increase in coil-turn structures, thus contributing to a complete inhibition of elastin calcification (246–248).

Supplementation of endogenous/exogenous molecules causing a calcium overload represents an efficient model to demonstrate the impact of inhibitors of calcification. For instance, a combination of vitamin D3 and nicotine (the so called VDN model) can induce a rapid hypercalcemia in rats, whereas the release of catecholamine is responsible for elastocalcinosis in large elastic arteries (249). These effects can be counteracted in rats by endothelin (250) and antioxidant molecules (251, 252).

Furthermore, the severe calcification induced in mice by overproduction of vitamin D3 can be downregulated by inhibitors of carbonic anhydrase (i.e, acetazolamide) since extracellular pH has a profound effect on calcium and phosphate solubility, which is enhanced by acidification and decreased by alkalinization (229).

An additional interesting *in vivo* model has been proposed by Basalyga and coworkers (167) who used a vascular injury model based on a single periarterial application of CaCl_2_ solution, which lead to calcium accumulation within elastic fibres and to elastin degradation, further highlighting the role of MMPs as key mediators of the calcification process. Consistently, MMP-deficient mice do not exhibit elastin fragmentation or vascular calcification.

Medial arterial calcification set up in the rat model was used to investigate antibody-target nanoparticles as a potential therapeutic strategy. In particular, chelating agents (e.g., EDTA) have been combined with human serum albumin-based nanoparticles which were functionalized with antibodies that selectively bind to specific epitopes exposed by degraded elastin. This treatment was able to reduce vascular calcification and improve arterial elasticity without side effects in bone or mineral metabolism (253, 254).

Although characterized by their cost-effectiveness, easy maintenance and manipulation and well-established methodologies, a limit of the models described so far is represented by physiological differences in heart rate, blood pressure and metabolism compared to humans. Therefore, large animal model of vascular calcification, such as pigs, can be used more efficiently in translational research and for testing of interventional devices, since they better mimic human cardiovascular physiology and vascular anatomy (255).

Since all models do not completely overlap what is occurring in humans, rare genetic diseases represent unique models for the discovery of fundamental and novel biological mechanisms. Indeed, monogenic defects, their penetrance in early life, and the expanding collection of human mutations and phenotypes make rare diseases ideal targets for exploring evolutionarily conserved biological mechanisms whose alterations may cause also common human diseases. However, also in this case there are some limits, such as the genetic heterogeneity among individuals, and the fact that human mutations can be exceedingly rare, thus preventing large-scale studies. Indeed, cardiovascular calcification is also a complication of several genetic diseases such as Generalized arterial calcification of infancy (GACI) (256), *Pseudoxanthoma elasticum* (PXE) (257), Singleton-Merten Syndrome (SGMRT) (258), Hutchinson-Gilford Progeria (HGPS) (259), calcification of joints and arteries (CALJA) (260), idiopathic basal ganglia calcification (261), as well as of chronic disease as in atherosclerosis, diabetes and chronic kidney disease (4, 262). Interestingly, in all these diseases, the causative gene is not directly related to elastin or to elastic fibre's components although, in most cases, elastic fibres are the preferential target of mineral deposition and therefore they may represent valuable models to deepen the knowledge on ectopic calcification. Indeed, a general unifying traits are represented by trans-differentiation of mesenchymal cells towards a pro-osteogenic phenotype (10, 263–266), increased expression and activity of tissue-nonspecific alkaline phosphatase (TNAP) and mitochondrial dysfunction, leading to oxidative stress and reduced availability of calcification inhibitors such as extracellular inorganic pyrophosphate (PPi) released upon activation of ectonucleotide pyrophosphatase/phosphodiesterase (ENPP1) and transported by transmembrane ankylosis protein (ANKH) (239, 267–270) (Figure 4).

## 8 Conclusions

Elastic fibres, and especially degraded elastin, play a critical role in the development and progression of calcification with several clinical implications for the cardiovascular tissues. This is due to the capabilities of elastic fibres to sequester Ca^2+^ ions fostering the formation of Ca^2+^-based mineralized structures that alter tissue elasticity and signalling pathways mediated by cell and cell-matrix interactions. Therefore, changes in the structure and composition of the ECM contribute to develop a suitable environment further triggering and promoting mineralization. Within this context, matrix components, whose amount and physico-chemical characteristics undergo site-specific age-related changes, modulate mesenchymal cell behaviour towards an osteogenic phenotype and regulate the different susceptibility of soft connective tissues to mineral deposition.

Both *in vitro* and *in vivo* models are essential for studying mineral composition and calcification mechanisms. I*n vitro* systems, in particular, enable to analyse the rate of mineral formation and its temporal evolution as well as the influence of regulatory factors. *In vivo* models, mimicking human diseases, offer insights into the role of genetic, biochemical, and environmental factors on elastin calcification and its inhibition.
